# Human α2‐Macroglobulin: Architecture, Mechanisms, and Functional Implications

**DOI:** 10.1096/fj.202503420R

**Published:** 2026-04-11

**Authors:** Pietro de Carvalho Andrade, Tales Alexandre Costa‐Silva, Gisele Monteiro

**Affiliations:** ^1^ Department of Biochemical and Pharmaceutical Technology, School of Pharmaceutical Sciences University of São Paulo São Paulo Brazil; ^2^ Center for Natural and Human Sciences Federal University of ABC Santo André SP Brazil

**Keywords:** alpha 2 macroglobulin, molecular mechanisms, plasma protein, protease inhibitor, proteinase

## Abstract

α2‐Macroglobulin (α2M) is a large glycoprotein and one of the most abundant proteins found in the plasma of vertebrates and in the hemolymph of invertebrates. It performs several important functions in the innate immune system, serving as a pan‐protease inhibitor and contributing to essential processes, such as cytokine and hormone transport. It also triggers diverse cellular responses, critical for the functioning of both eukaryotic and some prokaryotic biological systems. Here, we focus on the human α2‐macroglobulin (hα2M) molecule, compiling relevant information from recent studies on its structure and how this relates to its unique protease capture mechanism. In addition, we summarize other findings accumulated over 50 years of literature on hα2M. We also discuss its distinctive electrophoretic behavior, its dimeric form, and its potential role in inflammatory environments, as well as the basic unit of hα2M for protease capture. We summarize some challenges encountered in hα2M structural studies, a distinctive mechanism of incorporation of non‐proteolytic ligands, and remarks and details on its purification and storage. Although the clinical use of hα2M is currently limited mainly to its role as a secondary biomarker for certain disorders, new therapeutic approaches have begun to emerge in initial studies over the past decade. The study of hα2M remains highly relevant for understanding unknown aspects of the innate immune system, developing new therapies, elucidating infection and inflammation processes, exploring potential links to mechanisms of cancer resistance, and advancing other fields critically important to translational and clinical research.

## Graphical abstract

Visual representation of human α2‐macroglobulin in its native and induced conformations. General functions associated with the native form (green), the induced form (white), and both conformations (light red) are indicated.

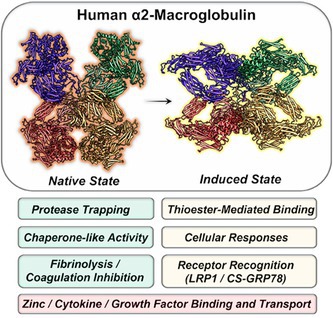

**Abbreviations**
α1I3: alpha‐1‐inhibitor‐3
α2MF: alpha‐2‐macroglobulin superfamily
α2ML1: alpha‐2‐macroglobulin‐like 1
α2Moo: alpha‐2‐macroglobulin from *Xenopus laevis* eggs (ovomacroglobulin)
<hα2M>: transient “nascent” intermediate of human alpha‐2‐macroglobulin
bFGF: basic fibroblast growth factor
bisANS: 4,4′‐dianilino‐1,1′‐binaphthyl‐5,5′‐disulfonic acid dipotassium salt
BR: bait region
BRD: bait region domain
C3, C4, C5: complement components 3, 4, and 5
CD109: cell surface antigen CD109
CH_3_NH_2_
: methylamine (MA)
CPAMD8: C3‐ and PZP‐like alpha‐2‐macroglobulin domain‐containing protein 8
cryo‐EM: cryogenic electron microscopy
CS‐GRP78: cell surface glucose‐regulated protein 78
CUB: complement C1r/C1s, Uegf, Bmp1 domain
Cys: cysteine (amino acid residue)
DNPSCN: 2,4‐dinitrophenyl thiocyanate ester
DTT: dithiothreitol
FAC: fibronectin–aggrecan complex
FGF1, FGF2, FGF4, FGF6: fibroblast growth factors
FP/NF: frozen fresh plasma/non‐frozen fresh plasma
GRP78: glucose‐regulated protein 78
HSA: human serum albumin
HSP70: heat shock protein 70
hα2M: human alpha‐2‐macroglobulin
hα2M*: induced human alpha‐2‐macroglobulin
hα2M–NH_3_
: amine induced human alpha‐2‐macroglobulin
IFN‐γ: interferon gamma
IgA/IgM: immunoglobulins A and M
IL‐1β, IL‐2, IL‐4, IL‐6, IL‐8, IL‐18: interleukins
IMAC/IEC: immobilized metal affinity chromatography/ion exchange chromatography
JAK: Janus Kinase
kDa: kilo dalton
LNK: linker region
LRP1: low‐density lipoprotein receptor‐related protein 1
MG 1–7: macroglobulin‐like domains 1–7
MHC: major histocompatibility complex
MPO: myeloperoxidase
NaOCl/NaSCN: sodium hypochlorite/sodium thiocyanate
NGF‐β: nerve growth factor beta
NH_3_/(NH_4_)_2_SO_4_
: ammonia/ammonium sulfate
PAGE: polyacrylamide gel electrophoresis
PDGF/PDGF‐BB: platelet‐derived growth factor/‐BB
PEG: polyethylene glycol
PZP: pregnancy zone protein
RBD: receptor binding domain
SDS: sodium dodecyl sulfate
STAT3: signal transducer and activator of transcription 3
TED: thioester domain
TEP: thioester‐containing protein
TGF‐β1/TGF‐β2: transforming growth factor beta 1/2
TNF‐α: tumor necrosis factor alpha
VEGF: vascular endothelial growth factor

## 1 Introduction

First isolated nearly 70 years ago, human α2‐macroglobulin (hα2M) is among the most abundant proteins found in plasma [1, 2, 3, 4, 5]. It performs several functions related to the innate immune system, primarily in the capture of proteases [3, 4, 6, 7, 8]. In the early 1970s, Barrett and Starkey proposed a unique mechanism underlying protease inhibition that contradicted the specificity and mechanistic rules established for other restrict peptidase inhibitors [1, 5]. A molecule capable of inhibiting all four classes of proteases through a bait region (BR) and spatial trapping, while preserving the ability of the trapped protease to cleave small substrates, but not large substrates, seemed somewhat counterintuitive even to the authors themselves [5]. However, in subsequent years, after extensive studies, the “trap hypothesis” remained practically unchanged, with the addition of information regarding a covalent bond—not essential for capture, as demonstrated by Barrett's group [5, 9]—to proteins trapped via an intrachain thioester present in members of the thioester‐containing protein (TEP) family [5].

This proposed mechanism of action is one of the most well‐established in the field of protein science, and no other study in the last 50 years has come near to refuting this idea. Nevertheless, due to the limitations of crystallographic techniques, the exact tetrameric structure and how the multiple domains of hα2M participate in the mechanism remain somewhat speculative [4, 10, 11, 12]. At the beginning of this decade, new cryo‐electron microscopy (cryo‐EM) models of the hα2M molecule in its native and induced forms were obtained [10, 11, 13, 14], providing a better understanding of the relationship between the hα2M domains and the capture mechanism. In addition, new speculations about the BR, which is internalized and difficult to visualize, and the possible trigger—the “plug‐in‐channel hypothesis”—that initiates the entire conformational change and trapping of proteases by hα2M were proposed [13, 15, 16].

In summary, the hα2M protease capture mechanism relies on steric entrapment without enzymatic activity. Through its large central cavity and other lateral holes, acting as a molecular sieve, access is provided to a BR within each of the four monomers. Protease cleavage of the BR initiates the mechanism and triggers a major conformational change in the affected monomer. This change propagates to the other monomers, thereby trapping the substrate and inducing a global rearrangement of the hα2M molecule. During this transition, an internal thioester bond is exposed and can covalently link to the protease. Moreover, the receptor binding domain (RBD), previously internalized, becomes exposed on the molecular surface. It can then interact with cell‐surface receptors, including low‐density lipoprotein receptor–related protein 1 (LRP1) and cell‐surface GRP78 (CS‐GRP78), promoting clearance of hα2M and its captured target [1, 3, 4, 5, 10, 11, 13, 14, 17]. The details of each step are discussed in subsequent sections.

Recent reviews describe the relevance of hα2M in inflammation and its roles in hemostasis, immunity, and infections, highlighting its biological structure and function, the conserved multifunctionality of α2M homologues, and its role as a “systemic physiological guardian” [4, 7, 8, 18]. In this review, we discuss new findings of this decade in the field of hα2M, providing relevant information on its architecture, structural and mechanistic aspects, its dimeric state, concepts related to purification and storage, as well as its functions reported in the literature. With the aim of assisting both new readers and experts in the field, we reinforce some established concepts and discuss ideas that may have been overlooked during these nearly 60 years of extensive studies on hα2M.

## 2 Discovery, Functions, and Evolutionary Homologues of hα2M

Human α2‐Macroglobulin (hα2M) was first described in 1946, when it was identified during plasma fractionation by ethanol precipitation performed by Cohn and colleagues, who divided plasma into five fractions [2, 4]. Most of hα2M is found in fraction III, along with immunoglobulins M (IgM) and A (IgA) [2]. However, it was not fully isolated until the following decade, around 1955, by the works of Jacobsson and colleagues, as well as Schultze and collaborators [5, 19]. Since the isolation of hα2M, numerous studies have been conducted to characterize its structure, mechanism of action, biological functions, and potential clinical applications.

hα2M is a large glycoprotein abundantly present in human plasma at a physiological concentration ranging from 2 to 4 mg/mL [4, 20]. It is primarily produced by hepatocytes in the mammalian liver, but is also synthesized by macrophages, fibroblasts, monocytes, and astrocytes [11, 21, 22, 23]. Considered an acute‐phase protein, its expression is positively regulated by the pro‐inflammatory cytokine interleukin‐6 (IL‐6), and it can also bind IL‐6, providing a negative feedback loop [18]. IL‐6–dependent Janus kinase (JAK) signaling activates signal transducer and activator of transcription 3 (STAT3), which binds the hα2M promoter and increases transcription [18, 24].

hα2M contributes predominantly to the innate immune system, with its primary function being the inhibition of proteases, including both endogenous and exogenous peptidases. hα2M is capable of inhibiting all four catalytic classes of peptidases—serine, cysteine, metallopeptidases, and aspartic proteases—making it a uniquely broad‐spectrum protease inhibitor [5, 11]. Therefore, hα2M serves as a key physiological regulator in the bloodstream, contributing to inflammation, host immune defense, and the response to infection [4, 7, 8]. It also regulates coagulation‐related peptidases (e.g., inhibition of thrombin, plasmin, factor Xa, kallikrein, urokinase, and activated protein C), thereby contributing to anticoagulant and antifibrinolytic effects that protect self‐tissues from proteolytic damage [7, 8, 18]. Beyond its well‐established role in protease capture, hα2M is involved in several other functions, such as the transport of cytokines, growth factors and hormones (e.g., FGF1, FGF2, FGF4, FGF6, IFN‐γ, IL‐1β, IL‐2, IL‐4, IL‐6, IL‐8, IL‐18, NGF‐β, PDGF, TGF‐β1, TGF‐β2, TNF‐α, VEGF) [4, 7, 8, 10, 25]. Additionally, it exhibits transglutaminase activity and binds zinc and copper ions (although these are not cofactors for its antiproteolytic activity [26]), while its holdase chaperone activity stabilizes misfolded proteins and facilitates their clearance, preventing aggregate formation under various in vivo conditions [11, 12, 26, 27, 28].

It exhibits anti‐inflammatory and anti‐apoptotic properties, and its dysregulation has been related with several diseases, including Alzheimer's disease, AIDS, diabetes, and cardiovascular disorders [19, 27, 29, 30]. Currently, the clinical use of hα2M is limited to its role as a secondary biomarker for some of these disorders described above (e.g., early marker of cardiac hypertrophy and myocardial infarction) [7, 30, 31]. However, based on its ability to capture proteases and bind pro‐inflammatory cytokines (e.g., TNF‐α, IL‐1β, and IL‐6), several potential clinical applications have been proposed [32, 33, 34, 35, 36, 37] and are discussed in Section 9. To date, no evidence supports the complete absence of hα2M, suggesting that it is essential for embryonic development [38]. Elevated concentrations are observed in pregnant women, possibly induced by estrogen [39, 40, 41]. Notably, high levels (e.g., as reported in pregnant smokers) have been associated with reduced embryonic development. Although, this relationship may be indirect and possibly reflects altered biomarker levels [41] as consequence of smokers' lifestyle.

As a key evolutionary component of the defense system—the immune system—α2‐Macroglobulin (α2M) has been conserved throughout species' evolution. Structural homologues of the human molecule are found in diverse organisms, including various metazoans, and even in prokaryotic cells, such as bacteria [4, 7]. A non‐definitive hypothesis proposes that bacterial α2M was acquired from metazoan hosts, as these proteins are primarily found in mutualistic and pathogenic Gram‐negative bacteria, and subsequently disseminated to other colonizing bacterial species through independent horizontal gene transfer events [4, 42].

In addition to interspecies homologues, hα2M shares significant structural similarity with other human innate immune molecules, particularly the complement proteins such as complement component 3–5 (C3, C4, C5). Together with pregnancy zone protein (PZP), alpha‐2‐macroglobulin‐like 1 (α2ML1), alpha‐1‐inhibitor‐3 (α1I3), cell surface antigen CD109 (CD109), and C3 and PZP‐like alpha‐2‐macroglobulin domain‐containing protein 8 (CPAMD8), hα2M comprises the TEP family, which is broadly classified into two groups: protease inhibitors and complement factors [4, 7, 8, 43, 44]. TEP family proteins have an evolutionary origin dating back over 600 million years—predating immunoglobulins. The earliest known α2M dates from approximately 500 million years ago in the hemolymph of the horseshoe crab *Limulus polyphemus* [44, 45]. One hypothesis suggests that the C3 protein originated from a gene duplication event of α2M, subsequently giving rise to the C4 and C5 proteins. Accordingly, all α2M homologues across different species are thought to have evolved from a common ancestor with a conserved core structure composed of eight homologous domains [8, 44, 46]. This hypothesis is supported by sequencing analyses and exon–intron organization studies, which reveal structural similarities between distinct α2M homologues and complement system proteins [4, 8, 43, 46]. After reviewing its discovery, functions, and homologues, we next examine the monomer‐to‐tetramer organization of hα2M and the challenges associated with producing reliable three‐dimensional models.

## 3 Monomer to Tetramer: Structural Complexity and Modeling Challenges of hα2M

### 3.1 Secondary and Tertiary Structure of the hα2M Monomer

**FIGURE 1:**
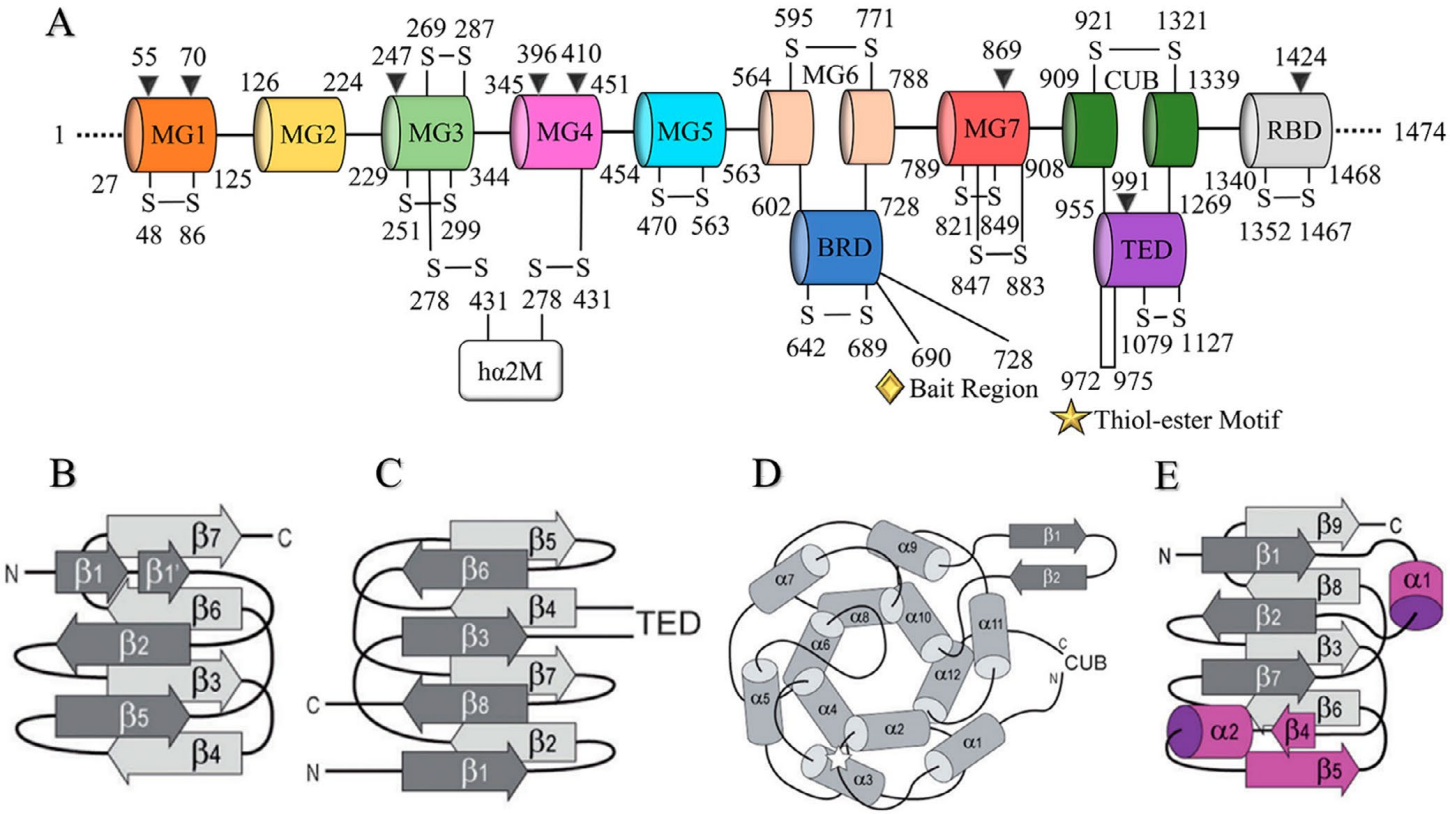
Structures of hα2M domains and secondary structures—(A) Domain arrangement of human hα2M. MG1–MG7, macroglobulin‐type domains 1–7; the intermolecular disulfide bond is formed between Cys^278^ in the MG3 domain and Cys^431^ in the MG4 domain of the adjacent monomer (indicated in the figure as hα2M); BRD, bait region domain; CUB, complement C1r/C1s, Uegf, Bmp1 domain; TED, thioester domain; RBD, receptor‐binding domain. The bait region (BR) is represented by a gold diamond, and the thioester site is represented by a gold star. Black triangles indicate glycosylation sites. Adapted from Huang et al. [10] and Luque et al. [11]. (B–E) Topology schemes with secondary‐structure elements. (B) MG domains; (C) CUB; (D) TED; (E) RBD. Elements distinguishing the RBD from MG domains are shown in magenta. Reproduced from Marrero et al. [14], 2012 Wiley‐VCH GmbH. All rights reserved.

hα2M is a 720‐kDa, glycosylated, homotetrameric protein with four identical 180‐kDa monomers. Each monomer contains 1451 amino acid residues (1474 including the signal peptide) and has 11 intramolecular and 2 intermolecular disulfide bonds. In addition, the monomers are organized into 11 domains (Figure 1A). hα2M is described as a “dimer of dimers” because each monomer forms two disulfide bonds with its neighbor, although the upper and lower dimers are not covalently linked [3, 4, 6, 7, 8, 11, 12, 13, 14, 19, 20].

The first seven domains, termed macroglobulin‐like domains (MG1–MG7), have structural function and are arranged as seven antiparallel β‐sandwiches composed of three‐ and four‐stranded sheets, each domain comprising approximately 110 residues (Figure 1B). Next, the complement C1r/C1s, Uegf, Bmp1 domain or complementary domain (CUB), linked to MG7, RBD, and TED, comprises 116 residues and consists of two β‐sheets, each formed by four antiparallel strands (Figure 1C). The thioester domain (TED) is linked between the β3 and β4 loops of the CUB domain via its N‐ and C‐termini. It contains 315 residues arranged in a disk‐like shape, consisting of six pairs of α‐helices surrounding a central axis; additionally, it features a β‐pleated sheet linking α‐helix 9 (α9) and α‐helix 10 (α10) (Figure 1D). The receptor binding domain (RBD), also known as MG8 due to its structural similarity, is located in the C‐terminal region of the hα2M monomer. The RBD is linked behind the CUB domain and is internalized in the native form of hα2M. It is composed of 129 residues and adopts a conformation similar to MG β‐sandwiches, featuring nine antiparallel strands arranged in four‐ and five‐stranded sheets (Figure 1E). Additionally, it contains an extra α‐helix and β‐α‐β unit (Figure 1E, shown in magenta) [4, 10, 11, 12, 13].

**FIGURE 2:**
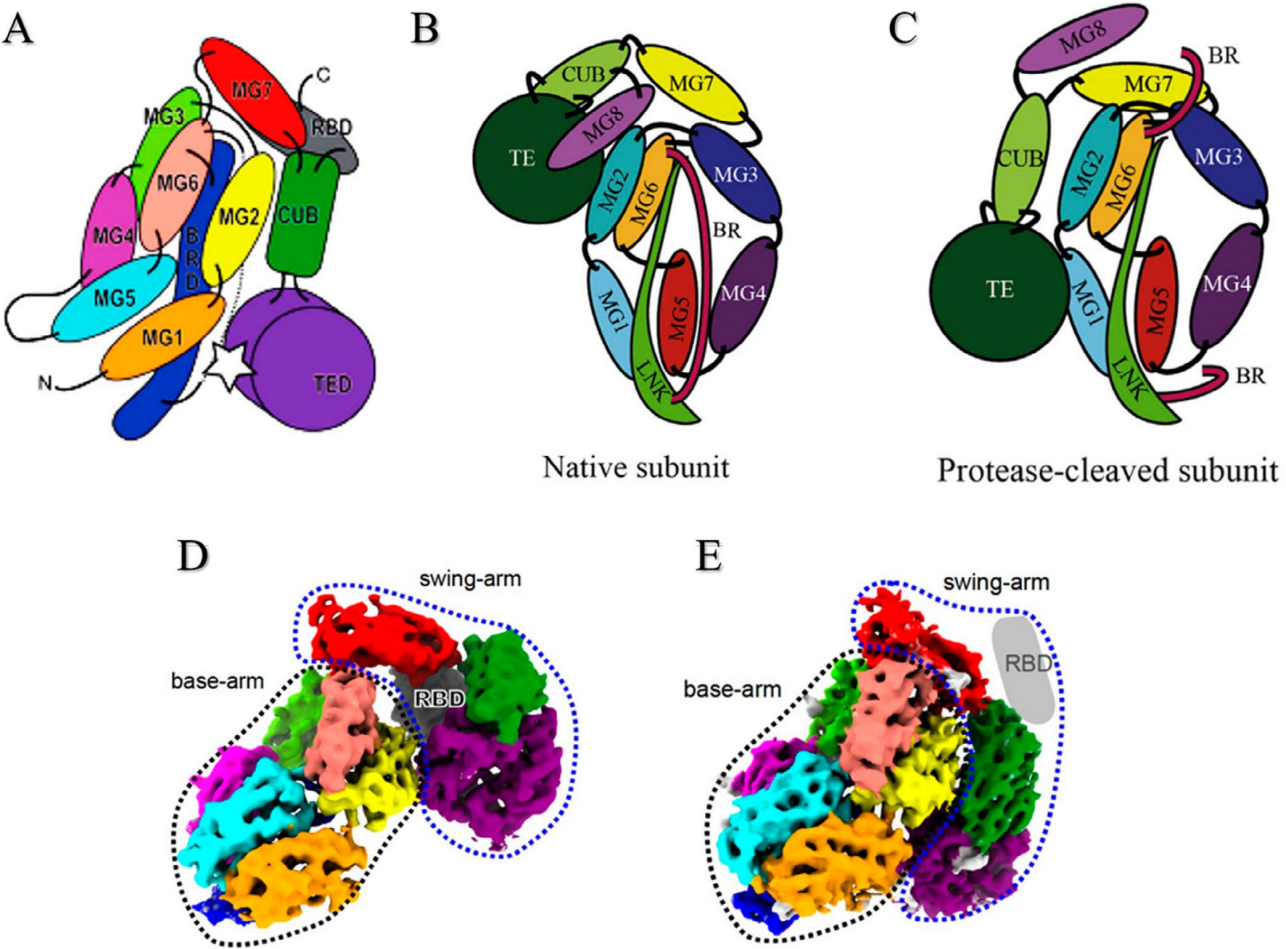
Schematic arrangement of the domains constituting the hα2M monomer. (A) Schematic arrangement of the domains in the hα2M monomer. The thioester site is indicated by a white star. The flexible bait region (BR) is depicted as a dashed line. Reproduced from Marrero et al. [14], 2012 Wiley‐VCH GmbH. All rights reserved. (B, C) Schematic representations of an hα2M subunit in its native and protease‐cleaved conformations, respectively. In all structurally characterized A2MF proteins, major conformational changes following bait region (BR) cleavage involve the MG7, CUB, TED (TE in the figure), and MG8 (RBD), whereas the MG1–6 domains and the linker (LNK) region form a mostly static framework. Reproduced from Harwood et al. [15] under CC BY 4.0 license: https://www.jbc.org/article/S0021‐9258(22)00672‐X/fulltext. (D, E) Density maps of the hα2M monomer in native (D) and induced (E) states, respectively. The expected position of the RBD is shown in gray. Reproduced from Huang et al. [10], with permission from Springer Nature.

Finally, the last domain, called the bait region domain (BRD), comprises 127 residues and is an extended and flexible portion inserted between the fourth and fifth β‐strands of MG6. It is located in the internal region of the monomer in its native form, occupying a large space of the vertical dimension of the internal cavity of the hα2M monomer, interacting with MG1, MG3, and MG5 (Figure 2A, shown in dark blue). It contains a 39‐residue bait amino acid sequence—also referred to as the bait region (BR)—which, upon cleavage by substrates, triggers a major conformational change in the hα2M homotetramer, directly associated with the protease capture and trapping mechanism. This mechanism will be described in detail in a later section [10, 11, 14].

Recent studies have divided the BRD into two portions: the linker region (LNK) and the BR (Figure 2B, shown in light green and outlined in red, respectively). The LNK is conserved in homologues of the α2M protein superfamily (α2MF). It is proposed to serve as the non‐covalent interaction interface between hα2M dimers and corresponds to the static portion of the BRD. In contrast, the mobile region, which interacts with LNK and MG6, is responsible for the major conformational change of hα2M upon destabilization of the BR (Figure 2C). This mechanistic hypothesis, termed the “plug‐in‐channel,” has not yet been fully confirmed. However, its studies initiated the understanding of the molecular trigger underlying the conformational rearrangement of hα2M during its protease capture mechanism [10, 13, 15].

The first six domains (MG1–MG6) form a compact, one‐and‐a‐half‐turn ellipsoidal superdomain shaped as a right‐handed superhelix and constitute the static region of the monomer known as the “base arm” (Figure 2A,D). The MG7 domain closes the superhelix and functions as a hinge connecting the CUB, TED, and RBD. Together, these three domains and MG7 form the mobile region of the monomer, collectively referred to as the “swing arm” (Figure 2D). Cleavage of the 39‐residue BRD region by a protease induces a major conformational change that moves the “swing arm,” exposing the RBD to the outer surface of the molecule (Figure 2E). Consequently, this induces significant changes in the angles, torsions, and distances between domains not only within the monomers but also throughout the entire hα2M tetramer. In contrast, the “base arm” remains mostly static during this conformational change [10, 11, 14]. Having dissected the secondary and tertiary structure of the hα2M monomer, we now consider its quaternary organization, as further refined by recent three‐dimensional studies.

### 3.2 Quaternary Structure: The Tetrameric Form

Recent works by Huang et al. [10] and Luque et al. [11] provide three‐dimensional models of the native and protease‐induced forms of the hα2M tetramer obtained by cryo‐EM technique. In addition, a third model, reported by Marrero et al. [14], presents the structure of hα2M in a methylamine (MA) induced form (hα2M‐MA), also resolved by cryo‐EM. MA is a small nucleophile that triggers a conformational change in the hα2M similar to that induced by proteases; this topic will be detailed in a later section [10, 11, 14]. These quaternary structures, along with the refined hα2M model based on the native C3 protein (Harwood et al. [13]), predict that native hα2M adopts a hollow cylindrical architecture with two open ends, as originally suggested by Feldman and colleagues in 1985 [47]. This contrasts with the model proposed by Sottrup‐Jensen [6], who envisioned the hα2M tetramer as forming a cross‐like shape composed of elongated, rod‐shaped monomers.

**FIGURE 3:**
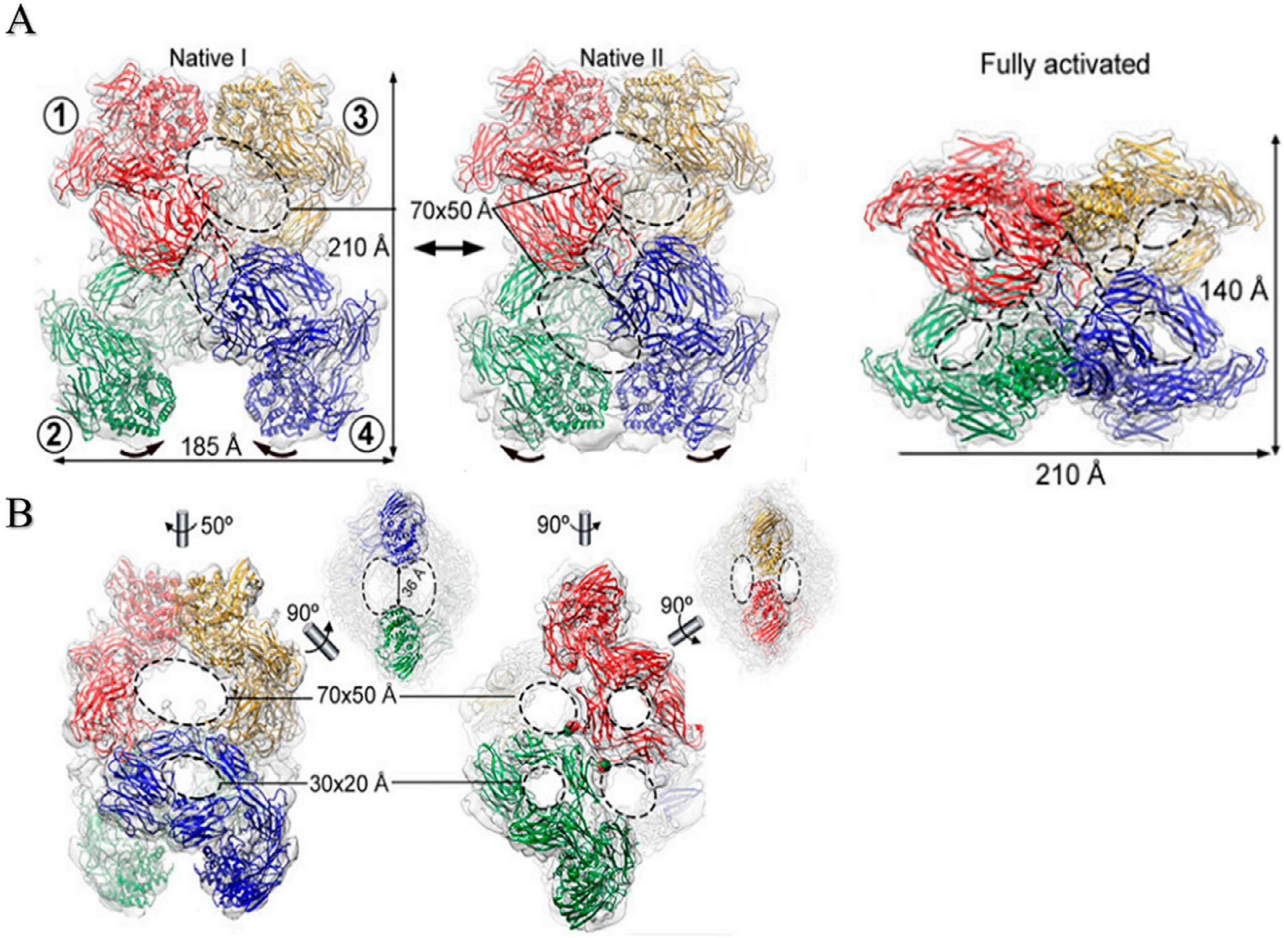
Cryo‐EM structures of human α2‐macroglobulin (hα2M) functional states. (A) Native and Induced States of the hα2M tetramer (hα2M) isolated from human plasma solved by cryo‐EM analysis, designated native I (left), native II (center), and activated (right). Protomer nomenclature: 1 (red), 2 (green), 3 (yellow), and 4 (blue). The red protomer forms disulfide bonds with the green protomer (disulfide‐linked), has a vicinal neighbor (yellow), and an opposite neighbor (blue). Native I and II states contain protomers in an expanded conformation and exist in equilibrium, with vicinal dimers adopting distal (native I) or proximal (native II) positions (indicated by curved arrows). The activated state (right, “H‐view”) shows all protomers in a compact conformation. Openings are marked by dashed ovals. Tethering loops of opposite protomers (1 and 4) are highlighted by a dashed rhombus. (B) Additional views of the native I (Left) and II (Right) complexes of (A). The latter highlights the disulfide‐linked residues between protomers 1 and 2 (red and green spheres). Dashed ellipses denote openings. (A, B) Reproduced from Luque et al. [11] under CC BY 4.0: https://www.pnas.org/doi/abs/10.1073/pnas.2200102119.

The native molecule exists in two conformations: one with a more open angle between monomers (Native I) and another with a more closed arrangement (Native II), with dimensions of 210 × 185 × 150 Å (Figure 3A, left and middle). In contrast, the conformation induced by MA (hα2M‐MA) or protease (hα2M‐protease) exhibits dimensions of 140 × 210 × 140 Å (Figure 3A, right) [10, 11, 14]. Intermediate models with one, two, or three “closed” monomers have also been reported [10, 11]. As mentioned, in addition to its hollow cylindrical structure (Figure 3A), hα2M exhibits multiple side openings of varying sizes (Figure 3B). The native states contain four large and four small apertures measuring approximately 70 × 50 Å and 30 × 20 Å, respectively. In contrast, the induced form displays 12 smaller openings of about 30 × 40 Å [11, 13]. These lateral openings, even in the hα2M‐MA, may account for protease incorporation following prolonged in vitro incubation [48, 49], as the BR within the BRD remains intact throughout this transformation, thereby permitting entrapment of proteases capable of accessing the BR. However, several studies have proposed that hα2M‐MA adopts a “closed” conformation and completely loses its antiproteolytic activity [50].

This aperture supports the role of hα2M as a “molecular sieve” in the protein‐rich plasma environment and corroborates the hypotheses proposed by Barret [3] (Figure 3B). According to this model, proteases not captured by hα2M may be (i) too large, resulting in steric hindrance that prevents access to the internal BR; (ii) very small (< 10 kDa), allowing them to pass through the cavity‐filled structure without interaction; or (iii) possessing limited proteolytic activity, insufficient to cleave the 39‐residue BR [3].

The three‐dimensional structures of hα2M–protease and hα2M–MA exhibit some structural differences, as demonstrated by the superimposed models reported by Marrero et al. [14] for hα2M–MA and Huang et al. [10] for hα2M–protease. First, these differences are primarily due to the higher density of the protease substrate within the “prey chamber,” which is absent in hα2M–MA—a concept that will be demonstrated in the following section. Second, the orientation of the RBD differs between the two induced forms, appearing at a lower position when induced by small primary amines such as methylamine. This difference stems from distinct conformational change mechanisms; although both represent induced states of hα2M, the conformational change of the “swing arm” occurs differently, as will be discussed in a later section [10, 12, 13]. With the quaternary hα2M tetramer resolved by recent high‐resolution models, the reasons these representations emerged only in recent decades will now be addressed.

### 3.3 Historical Challenges in Modeling the Three‐Dimensional Structure of hα2M

The theoretical mechanism of hα2M conformational change in protease capture, proposed by Barrett and Starkey, has been established for over 50 years, yet the precise behavior of individual domains and the three‐dimensional reorganization of the tetrameric molecule remain subjects of ongoing debate [4, 5, 10, 11, 12]. This challenge stems from the large size and intrinsic flexibility of hα2M, as well as the lack of recombinant expression systems, described only recently, capable of producing homogeneous, functional protein at scale [11, 51, 52]. Moreover, the internalized BRD is difficult to visualize, even in high‐resolution cryo‐EM models [53]. Additionally, structural heterogeneity in samples purified from natural sources further complicates analysis [11].

The first low‐resolution structural models of hα2M were obtained in 1968 using negative staining and early cryo‐EM techniques [4]. Subsequent advances, including cryo‐EM with three‐dimensional image reconstruction and low‐resolution X‐ray crystallography, allowed more detailed analyses. Nevertheless, early observations were largely limited to homologous models derived from the native complement C3 protein, fitted to low‐resolution cryo‐EM maps and the structural basis of the protease‐trap mechanism remained largely speculative [4, 11]. The only available induced conformation model, by Marrero et al. [14], used the small nucleophile MA and revealed a structure similar but not identical to that induced by proteases [10, 11, 12, 14, 15].

In 2022, two high‐resolution cryo‐EM studies (Luque et al. [11] and Huang et al. [10]) provided refined three‐dimensional structures of hα2M, spanning the native state to the fully protease‐induced state (hα2M–protease) [10, 11]. In addition to these models, Harwood et al. [13] presented a refined model based on the native C3 protein; Nielsen et al. [16] reported the cryo‐EM structure of α2ML1, an α2MF homologue; and Arimura and Funabiki (2021) published the structure of the *Xenopus laevis egg* homologue (α2Moo or ovomacroglobulin) [12]. These studies have provided a clearer understanding of hα2M's structure and monomer dynamics change during the protease capture mechanism, confirming several hypotheses proposed in mechanistic and structural investigations from the 1970s through the 1990s, which were often merely speculative.

## 4 Protease‐Trapping Mechanism

### 4.1 Conformational Dynamics, “Prey Chamber,” and Protease Capture Stoichiometry

**FIGURE 4:**
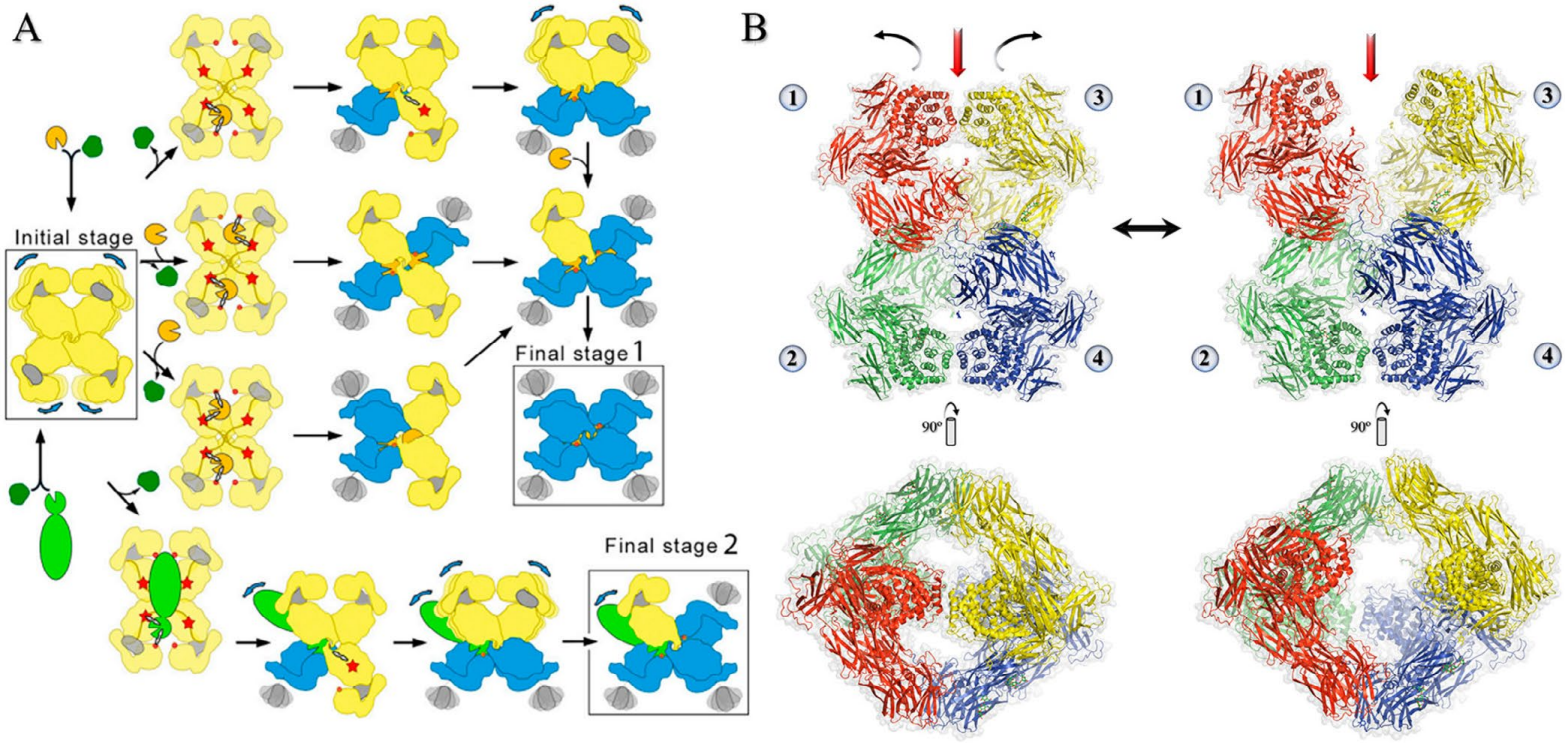
Mechanism of irreversible protease inhibition by hα2M. (A) Initial stage: Native hα2M (yellow, expanded subunits) acts as a molecular sieve for plasma proteins (dark green, light green, and gold). Only proteases (gold and light green) are entrapped within the internal cavity of hα2M following cleavage of the bait region (BR) (red star). Small proteases may be entrapped at a 2:1 stoichiometry (gold), or a single large protease at 1:1 stoichiometry (light green). The thioester bond (red dot) becomes exposed during the conformational change triggered by BR cleavage, enabling covalent bonding with the trapped protease. This conformational transition converts the protomer from an expanded (yellow) to a compact (blue) state, exposing the flexible receptor‐binding domain (RBD, gray) on the surface, facilitating binding to cells expressing LRP1 and/or CS‐GRP78 receptors. Since the trapped protease remains catalytically active inside the chamber, it can continue cleaving additional protomers, producing distinct intermediates and ultimately leading to the fully compact stage 1 for one or two small proteases. Blue arrows indicate domain flexibility. Reproduced from Luque et al. [11] under CC BY 4.0: https://www.pnas.org/doi/abs/10.1073/pnas.2200102119. (B) Native hα2M tetramer in its flexible conformations, shown in “H‐view” (PDB ID: 7O7M, left; PDB ID: 7O7L, right). Red arrows indicate the entrance to the central cavity; curved black arrows denote structural flexibility; opposing black arrows indicate the equilibrium between these conformations. (1) Reference protomer forming a covalent bond with protomer (2) via disulfide bond; this subunit interacts non‐covalently with protomer (3) (vicinal) and protomer (4) (opposite). Bottom left and right panels show 90° rotations along the *Y*‐axis of these structures, respectively, illustrating the opening of the central cavity between two vicinal protomers. Visualized with PyMOL v3.1.3 (Schrödinger LLC).

Following the structural characterization of hα2M, the molecular mechanism of protease capture is discussed. Nearly half a century ago, Barrett and Starkey proposed the hα2M protease trap mechanism, which has since been investigated extensively through various studies and experiments aimed at validating or refuting the hypothesis. However, despite the discovery of additional features, such as the covalent thioester bond, Barrett and Starkey's hypothesis has remained largely unchanged and stands as one of the most enduring models in the field of protein mechanism characterization [1, 5]. The protease capture mechanism has been termed the “Venus flytrap” in hα2M, analogous to the carnivorous plant's trapping of flies, whereas some monomeric homologues employ a “snap‐trap” mechanism [4, 8, 14]. Briefly, protease capture by hα2M and subsequent clearance occurs through a sequence of steps (Figure 4A): (i) proteolytic cleavage of the BR within the BRD; (ii) conformational change of hα2M; (iii) cleavage of the thioester bond in the internal TED and covalent attachment to the protease; (iv) exposure of the previously buried RBD; and (v) clearance of the complex by cells expressing LRP1 or CS‐GRP78 receptors [4, 7, 10, 11, 14].

**FIGURE 5:**
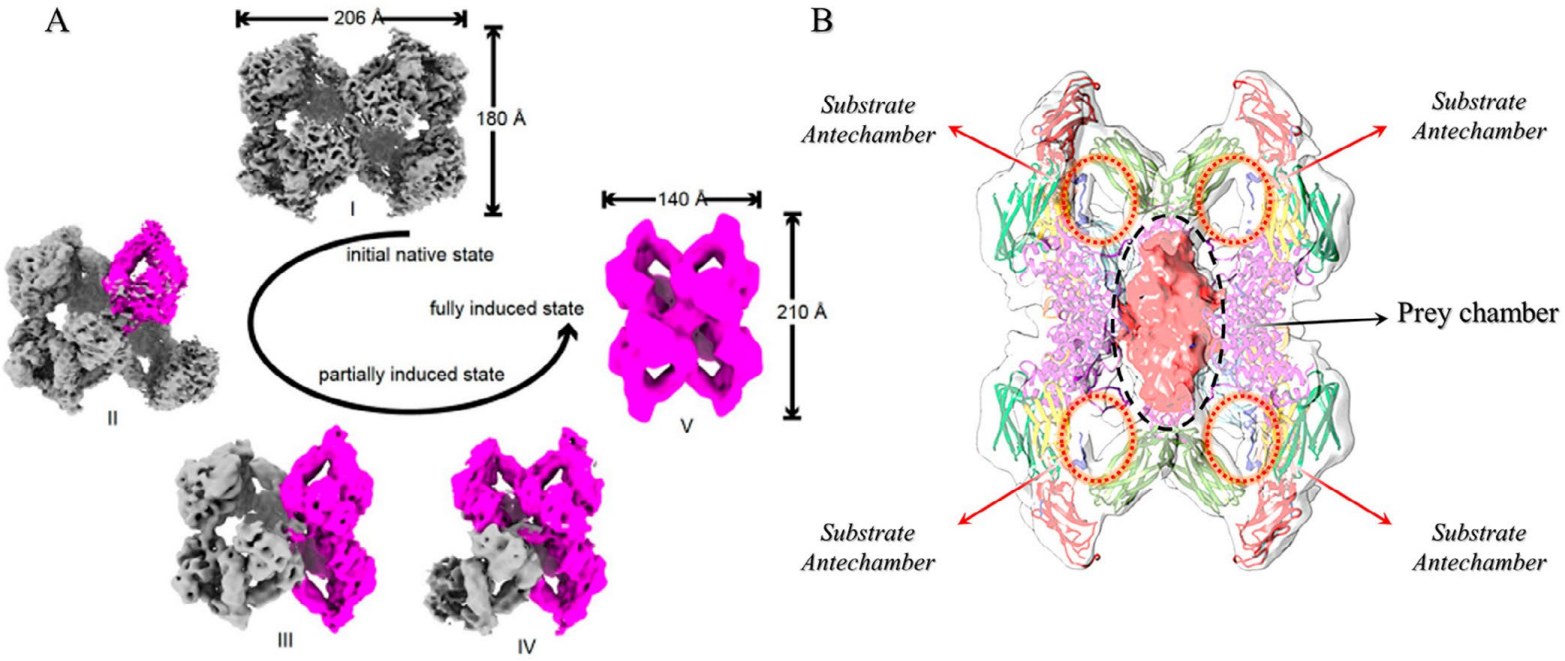
Dynamic transformation of human α2‐macroglobulin: Cryo‐EM structures of native, partial, and fully induced states and “Prey Chamber.” (A) Cryo‐EM structures of hα2M purified from human plasma showing different conformational states, including the native state (I), partially induced intermediate states (II, III, and IV), and the fully induced state (V). Two protomer types are colored in gray (native) and magenta (induced). Reproduced from Huang et al. [10], with permission from Springer Nature. (B) Intrinsically activated (hα2M) from plasma. The front half of the map (transparent surface) was removed to visualize the heterogeneous proteinase density (red). The black circle indicates the “prey chamber” region, and the red circles indicate the “substrate antechambers” regions. Reproduced from Luque et al. [11], with modifications, under a CC BY 4.0 license: https://www.pnas.org/doi/abs/10.1073/pnas.2200102119.

For a better understanding of the protease capture mechanism, it is essential to elucidate the spatial arrangement of the monomers. A reference monomer forms a non‐covalent vicinal dimer with an adjacent monomer, a covalent disulfide‐linked dimer with another subunit, and interacts with an opposite monomer positioned across from it (Figure 4B) [11, 13]. The monomer referred to here as the reference monomer forms a protease capture region together with its vicinal monomer. Each hα2M monomer contains a BR (a 39‐residue segment within the BRD); thus, the tetrameric molecule possesses four BR, but only two interconnected protease capture regions (Figure 4B) [4, 7, 13, 14]. The recent studies conclude that hα2M adopts intermediate conformations, with monomers induced independently of their disulfide‐linked counterparts upon capture of a small protease, as shown by intermediates with one to three induced monomers and fully induced species capturing a single trypsin [10, 11, 12]. This contrasts with the other models proposed, which hypothesized simultaneous transformation of all four hα2M monomers [10, 11, 12, 13, 14]. Upon interaction with proteases, hydrolysis of the BR by the substrate protease triggers a conformational change in this monomer; the proteolysis‐induced structural rearrangement in one monomer is transmitted to the other uncleaved monomers, resulting in four monomers in induced states (Figure 5A). During this step, a second fast‐acting protease, such as trypsin (trypsin–hα2M binding time: ~0.05 s), can reach the remaining regions of the opposite dimer bait, thus forming a 2:1 stoichiometry for small proteases relative to hα2M [3, 4, 6, 10] (Figure 4A).

This model is consistent with the protease capture mechanism proposed by Barrett and Starkey in 1973, as well as with multiple studies reporting that hα2M can capture two small proteases (2:1 stoichiometry; one per dimer—for example, trypsin, ~25 kDa) or a single large protease (1:1), such as plasmin (~90 kDa) [3, 4, 6, 7, 14, 19] (Figure 4A). This stoichiometry is now understood to be associated with the size of the internal cavity formed by the interaction of the four monomers during protease capture. This cavity, referred to as the “prey chamber,” accommodates the protease within the hα2M structure and has an estimated volume of ∼600 nm [3] in the native form (before capture) and ∼300 nm [3] in the induced form (after capture) [11]. In addition, this cavity is complemented by additional volumes contributed by the concave interior of each of the four monomers, referred to as the “substrate antechambers” (Figure 5B) [3, 4, 13, 14]. Owing to the considerable structural flexibility of the hα2M molecule, it is capable of accommodating proteases with diameters exceeding ~60 Å [12].

The presence of four BR in hα2M does not imply that each monomer independently possesses protease‐trapping capability [54], nor does it suggest that isolated disulfide‐linked dimers retain antiproteolytic activity independently [27, 55, 56]. As protease capture relies exclusively on the spatial entrapment of the substrate between two vicinal monomers, this indicates that the basic unit for the antiproteolytic activity of hα2M is a non‐covalently linked dimer [56] (Figures 4B and 5B), which will be discussed later. In addition, cleavage of the BR is required, meaning that the substrate protease must exhibit both catalytic activity and sufficient affinity toward the 39‐residue BR sequence [4, 7, 10, 11, 14, 15, 53]. As previously hypothesized based on the ability of hα2M to capture trypsin with acetylated lysine residues [57]—without requiring covalent thioester linkage—this concept was further substantiated by the recent study of Harwood et al. (2021) [51]. The authors generated a recombinant hα2M variant in which the 39‐residue BR was replaced with 13 repeats of the Gly‐Gly‐Ser sequence, a substrate sequence to which trypsin lacks affinity. This modified hα2M failed to capture trypsin, despite maintaining its native conformation and inducibility by primary amines [53].

### 4.2 Covalent Bond Formation via the Reactive Thioester (β‐Cysteinyl‐γ‐Glutamyl)

Following cleavage of the BR, the second stage of the mechanism involves covalent attachment of the protease to a reactive thioester bond, which is disrupted during the conformational change in hα2M triggered by proteolytic attack [4, 11, 12, 13, 15]. In monomeric and dimeric homologues, this constitutes the primary step in substrate protease capture, as shown for PZP and α1I3. When these TEP family proteins are incubated with small primary amines that disrupt the thioester bond, they entirely lose their antiproteolytic capacity. In contrast, tetrameric hα2M treated with primary amines retains the ability to bind proteases, though with slower kinetics [48, 49].

**FIGURE 6:**
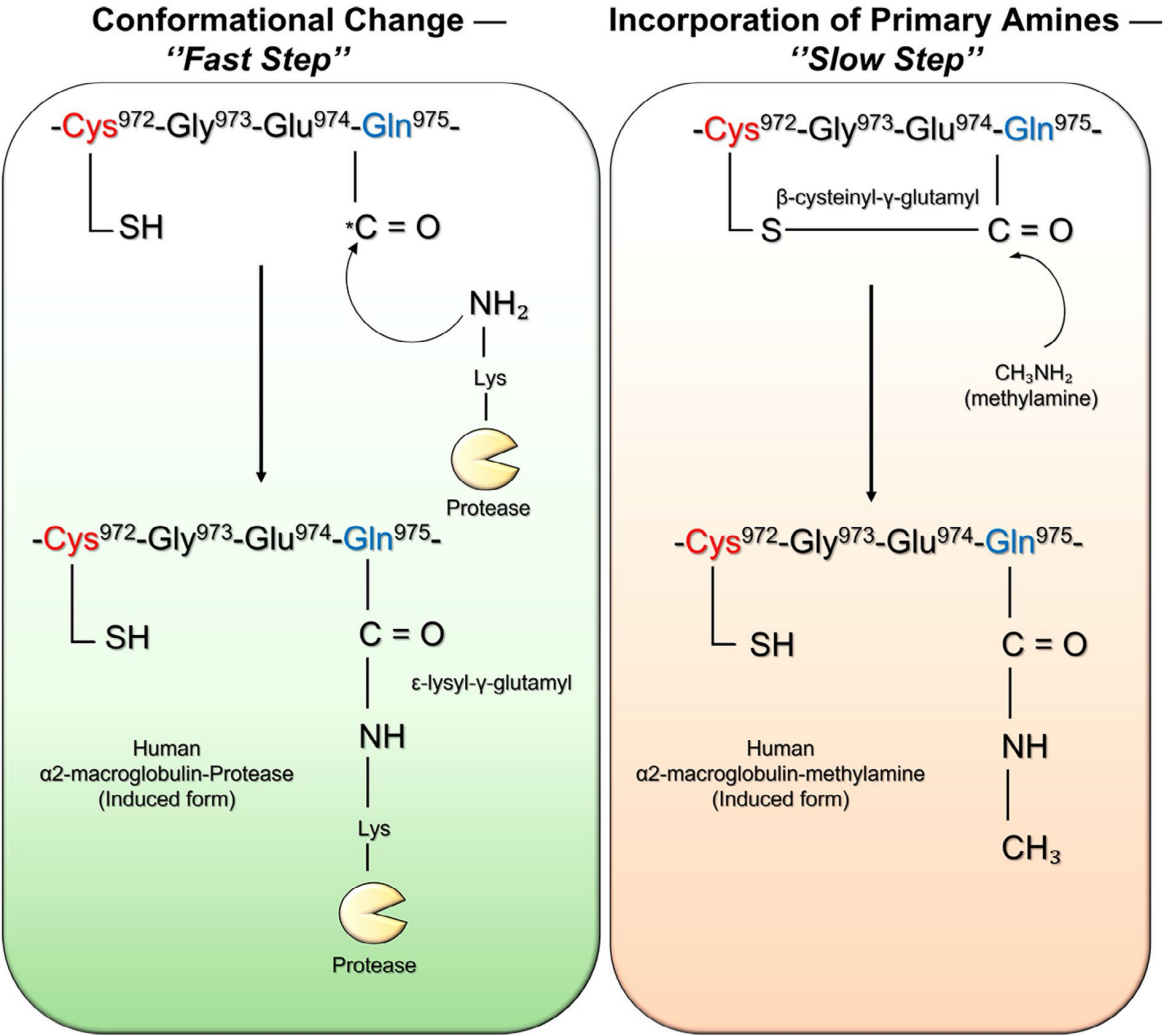
Mechanism of β‐cysteinyl γ‐glutamyl thioester cleavage. (Left) The thioester bond is rapidly cleaved after a conformational change triggered by proteolytic cleavage of the bait region (BR). The highly reactive γ‐glutamyl group of Gln^975^ then undergoes nucleophilic attack by surface‐exposed lysine amines of the entrapped protease, indicated by the curved black arrow. (Right) After primary amines penetrate the hydrophobic pocket in a slower step, they nucleophilically attack the γ‐glutamyl thioester bond. The cleavage of this bond and the disruption of the hydrophobic pocket also trigger a conformational change in the hα2M molecule. The nucleophilic attack is indicated by the curved black arrow. Adapted from Armstrong [58].

The thioester segment, composed of 15 atoms, forms a covalent bond between the sulfhydryl group (–SH) of Cys^949^ and the carbonyl group of Gln^952^, or to residues 972–975 (motif C ^972^‐G^973^‐E^974^‐Q ^975^) when accounting for the 23 amino acids of the signal peptide [6, 11, 14, 51]. This reactive bond is buried within the TED. Furthermore, this bond is shielded from hydrolysis by surrounding aromatic and hydrophobic residues within the TED and RBD, which form a protective hydrophobic pocket [4, 7, 12]. After cleavage of the thioester bond, the highly reactive carbonyl group of glutamine is attacked by nucleophilic amines from lysine residues in the protease, resulting in the formation of a covalently linked ε‐lysyl‐γ‐glutamyl adduct between hα2M and the substrate protease (Figure 6) [4, 7, 11, 59]. However, this interaction is not merely a simple anchoring event; protease molecules become covalently linked in distinct orientations relative to the hα2M structure and may form multiple covalent bonds, depending on their molecular size and spatial proximity to an additional thioester site on a neighboring monomer [6].

In addition to thioester cleavage triggered by hα2M conformational change, small primary amines can also induce thioester rupture, resulting in a structural transformation of the hα2M tetramer analogous to that observed during protease capture (Figure 6) [4, 6, 7, 11, 12, 19, 50]. Small molecules such as methylamine, ethylamine, and ammonium ion exhibit higher reactivity toward the thioester bond, whereas bulkier amines—such as n‐butylamine, isobutylamine, or dimethylamine—display reduced access to the reactive site due to steric hindrance imposed by the hydrophobic pocket [6, 59]. Furthermore, the incorporation of small primary amines depends not only on their molecular size but also on the pH of the reaction medium. Higher pH values enhance amine incorporation; however, pH levels above 9 may compromise the structural integrity of hα2M [6, 9].

The two modes of cleavage of the β‐cysteinyl‐γ‐glutamyl thioester bond—either via conformational change during protease capture or through primary amine attack (Figure 6)—result in the exposure of free thiol (‐SH) groups at Cys^949^. These groups can be titrated to assess bond stoichiometry or to analyze the native and induced conformations of each hα2M monomer [17]. The exposed thiol group can also react with molecules such as iodoacetamide, undergo thiocyanation by 2,4‐dinitrophenyl thiocyanate ester (DNPSCN), or bind to β‐aminopropionitrile, guanosine, imidazole, and thymidine [3, 6, 9, 60, 61]. Although both mechanisms induce similar structural changes in the hα2M tetramer, they differ mechanistically in the way they trigger the conformational transition and in the kinetics of the reaction [12, 13, 15]. Proteolysis triggers a rapid conformational change (< 15 s), whereas primary amine attack results in a slower transition occurring over minutes or even hours, depending on amine concentration (Figure 6) [6, 13, 50, 58].

In addition, the tertiary structures differ slightly. While protease attack causes collapse of the BRD and a conformational change triggered by the BR, as described in Section 3.1, attack by small amines appears to weaken the hydrophobic interactions between the RBD and TED [10, 15, 53]. In this case, polar groups would be generated in the previously hydrophobic environment that protects the β‐cysteinyl‐γ‐glutamyl bond, resulting in the release of RBD from TED, while the BR of the BRD remains intact [10, 11, 12, 15]. Despite these differences, both mechanisms result in exposure of the C‐terminal RBD and facilitate rapid clearance of the induced hα2M tetramer from the bloodstream [4, 7].

### 4.3 Receptor Binding Domain (RBD) Exposure and Clearance of Activated hα2M

Finally, the last step in the protease capture mechanism involves recognition of the exposed C‐terminal RBD region following conformational change of the hα2M tetramer. The RBD region binds cell surface ligands, including the low‐density lipoprotein receptor–related protein 1 (LRP‐1) and glucose‐regulated protein 78 (GRP78) [4, 8]. Induced forms of hα2M have a half‐life of less than 10 min in circulation, with much of this clearance mediated by hepatocytes and Kupffer cells; native hα2M does not interact with these receptors [4, 6, 7, 13, 62].

hα2M interaction with LRP1 depends on lysine 1393, while binding to cell surface GRP78 (CS‐GRP78) involves lysine 1397—positions 1370 and 1374 without the 23‐residue signal peptide [13, 63, 64]. Both residues lie within an α‐helix of the RBD and become exposed in the induced form of hα2M [8, 10, 64, 65]. In the native conformation, the RBD is buried inside the hydrophobic pocket, where it protects the β‐cysteinyl‐γ‐glutamyl thioester bond through interactions with residues Glu^223^ and Glu^197^ of the MG2 domain [10]. Recombinant expression of the RBD containing mutations at these two lysine residues abolished its ability to bind the receptor, indicating that they are critical for hα2M recognition by LRP1 and CS‐GRP78 [13, 63, 64, 66].

GRP78 is a member of the heat shock protein 70 (HSP70) family found in all eukaryotes on the membrane of the endoplasmic reticulum, but it can also be detected on the cell surface (CS‐GRP78) under conditions of overexpression, where it functions as a multifunctional receptor [67, 68, 69, 70, 71]. CS‐GRP78 is expressed at low levels per cell (~10 000) compared with LRP1 (~250 000 per cell) [71]. However, it has a higher affinity for hα2M [65, 67, 71], with a *K*_d_ of 50–100 pM, whereas LRP1 has a *K*_d_ of 1 to 10 nM for hα2M [65, 71, 72]; These two receptors are not cooperative with each other [72]. CS‐GRP78 is overexpressed in many cancer cell lines [65, 69, 70, 71, 72], and its binding to activated hα2M has been shown to have an insulin‐like effect, promoting pro‐proliferative, anti‐apoptotic, and pro‐migratory responses [65, 71, 72]. Induced hα2M can also promote trophoblastic cell fusion through CS‐GRP78 [68]. Additionally, CS‐GRP78 is expressed on the surface of differentiated macrophages but not on the surface of naive cells [71, 72].

These two receptor types enable induced hα2M to interact with diverse cell types, including fibroblasts, hepatocytes, adipocytes, syncytiotrophoblasts, astrocytes, monocytes, and macrophages. This interaction participates in various pathways and functions of hα2M described in Section 2 in a general manner—e.g., in macrophages, depending on the ligand and receptor‐mediated interaction, it can promote bacterial phagocytosis and reactive oxygen species (ROS) production via LRP1; enhance cell growth and migration through LRP1 and/or CS‐GRP78; trigger the release of inflammatory mediators via both receptors; and support antigen presentation via Major Histocompatibility Complex class I (MHC‐I) and class II (MHC‐II) [4, 7, 8, 10, 13]. These biological functions have been extensively described in recent reviews [4, 7, 8, 18]. The study of these pathways can expand our knowledge and provide new perspectives on mechanisms of the innate immune system, cellular responses, cancer research, and potential mechanisms of drug resistance. After addressing the detailed characterization of hα2M structure and protease capture, attention turns to its dimeric form, initially identified in vitro and later associated with in vivo oxidative stress and disease‐related contexts.

## 5 hα2M Dimers and Their Potential Role in Inflammation

The myeloperoxidase/H_2_O_2_/Cl^−^ system is primarily responsible for hypochlorite generation by innate immune defense cells in blood and synovial fluid [73, 74]. This oxidizing agent, produced at millimolar concentrations, acts against invading cells by inducing protein misfolding and aggregation and oxidative stress. However, this effect is nonspecific and also impacts host proteins [27, 73, 74]. The accumulation of misfolded proteins is associated with various acute and chronic inflammatory diseases and conditions, including Alzheimer's disease, type II diabetes, atherosclerosis, arthritis, emphysema, bacterial infections, Parkinson's disease, and others [27, 74, 75]. Studies have shown that hα2M, which is also active in inflammatory processes, is modified by hypochlorite, thereby regulating its primary functions of protease capture and cytokine transport [27, 73, 74, 75]. In addition, dimeric forms of hα2M were found in greater abundance in the serum of patients with diabetes compared to healthy controls, providing further evidence that hα2M dysregulation is indirectly associated with various diseases [76].

**FIGURE 7:**
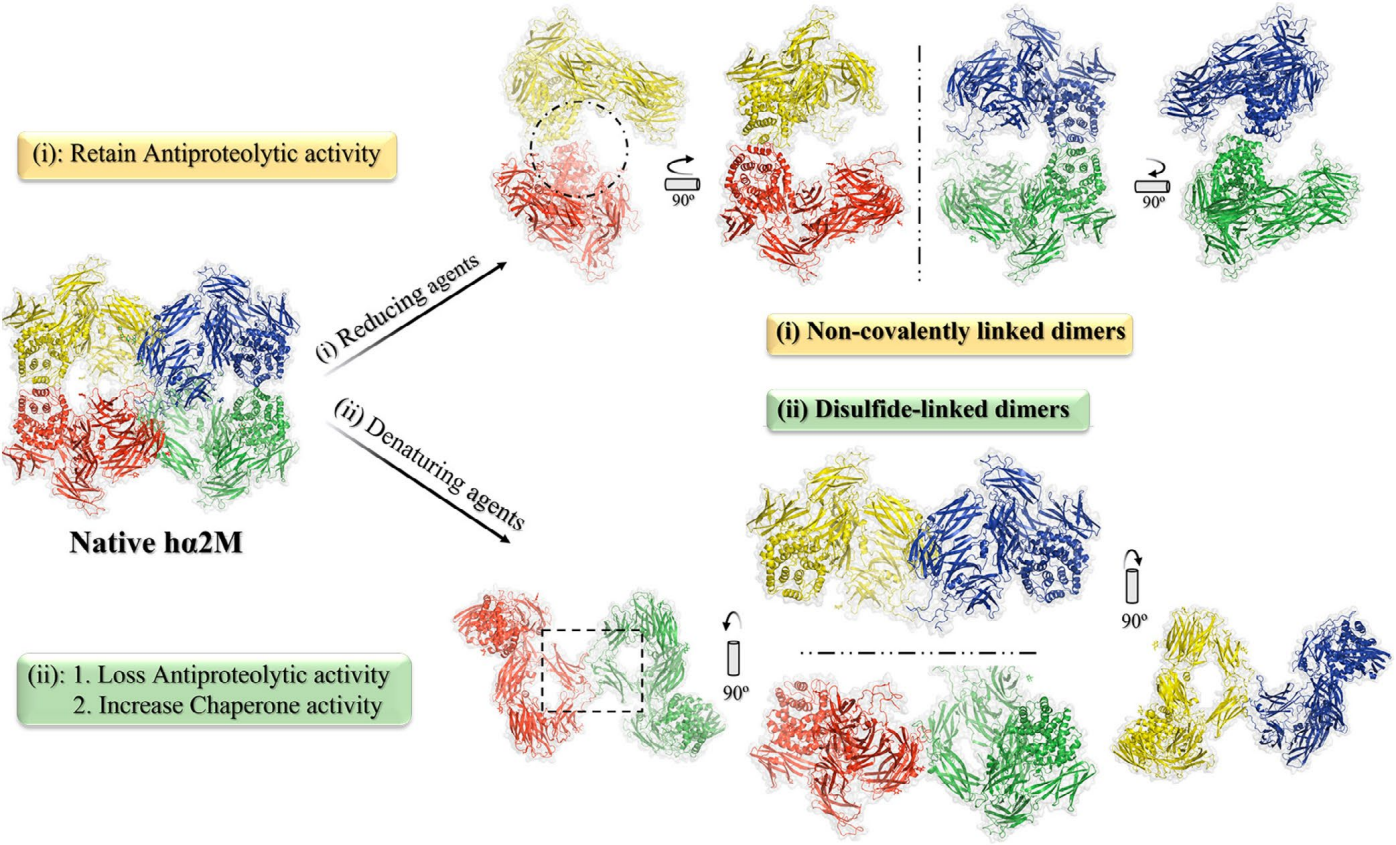
Disulfide‐linked and non‐covalently linked dimers of hα2M present different actions. (i) Non‐covalently linked dimers after treatment with reducing agents (top). (ii) Disulfide‐linked dimers after treatment with denaturing agents (bottom). hα2M as shown in “X‐view” (PDB ID: 7O7M). In both panels, the reference protomer (red) forms a covalent bond with protomer (green) via a disulfide bond and interacts non‐covalently with protomer (yellow) (vicinal) and protomer (blue) (opposite). The dashed circle in (i) indicates the “prey chamber” cavity for protease capture; the dashed line along the *Y*‐axis shows the separation of disulfide bonds. In (ii), the dashed square denotes the hydrophobic surface region of enhanced chaperone activity; the dashed line along the *X*‐axis shows the separation of non‐covalent interactions. Visualized with PyMOL v3.1.3 (Schrödinger LLC).

Native hα2M molecules exposed to hypochlorite tend to irreversibly dissociate into covalently linked disulfide‐bonded dimers, lose their ability to capture proteases, and expose receptor‐recognition binding sites [27, 55, 74]. However, this dissociation of hα2M into dimers markedly increases its holdase‐type chaperone activity (without ATP consumption), stabilizing misfolded proteins and preventing aggregation of a wide range of proteins in vitro (Figure 7) [27, 73, 75]. In addition, oxidized hα2M dimers preferentially bind to cytokines and proinflammatory factors (TNF‐α, IL‐2, IL‐6, and bFGF) and decrease the transport of anti‐inflammatory factors (PDGF, TGF‐β1, TGF‐β2, and NGF) compared to the native molecule, suggesting negative regulation of the acute inflammatory response through clearance of these complexes [77]. These findings led to the proposition that, in a hypochlorite‐mediated inflammatory environment with a high protease load, both hα2M species function via slightly different pathways: (i) native hα2M, which captures and eliminates proteases by binding to LRP1 receptors; and (ii) hypochlorite‐induced dimeric hα2M, which is unable to capture proteases but binds misfolded proteins with enhanced affinity and whose clearance is mediated by the LRP1 receptor [27].

This increased chaperone activity of hα2M dimers is primarily due to the exposure of a hydrophobic surface that was previously buried within the molecule in its tetrameric form [27, 73] (Figure 7). Studies by Wyatt et al. [72] demonstrated that hα2M treated with sodium thiocyanate (NaSCN), a non‐oxidizing agent capable of inducing the dimeric form, also exhibited increased chaperone activity, relating this activity exclusively to the exposure of hydrophobic surfaces rather than to oxidation itself. In addition, the study reports that the dimers do not undergo self‐aggregation and remain stable for months at 4°C [73]. Other oxidizing agents, besides hypochlorite, were less efficient at dimerizing hα2M; however, oxidation by hypochlorite and other agents induced modifications in lysine, tryptophan, tyrosine, methionine, and cysteine residues, as well as alterations in the secondary structure of the hα2M molecule [73, 74]. This degradation of amino acids is likely the reason why hα2M treated with MA (hα2M‐MA) and oxidized by hypochlorite loses its ability to bind LRP1 receptors, due to amino acid degradation in the exposed RBD of the induced molecule; however, oxidized hα2M‐MA species retain the ability to bind CS‐GRP78—formerly referred to as the signaling receptor or a high‐affinity binding site—and to activate signal transduction in macrophages [78]. Although oxidation can degrade the solvent‐exposed amino acid residues mentioned above, previous studies have shown that hypochlorite‐induced oxidation does not affect the integrity of the hα2M BR or thioester bond [74]. This loss of antiproteolytic activity was attributed instead to structural degradation of oxidized disulfide‐linked dimers, which become unable to entrap proteases. In contrast, dimers formed using bis(sulfosuccinimidyl) suberate (BS [3]), a non‐oxidizing cross‐linker, retained antiproteolytic capacity (Figure 7) [73, 74].

As little as 50 μM sodium hypochlorite (NaOCl) is sufficient to convert 90% of hα2M into dimers in vitro [73]. In addition to this agent and others mentioned above, hα2M dimerization can occur reversibly or irreversibly depending on the agent. Examples include acidic pH [79], dithiothreitol (DTT) [54, 56], urea [50, 56, 73, 80], sodium dodecyl sulfate (SDS), lyophilization [81], freezing [81], high concentrations of zinc and other divalent cations [26], as well as other destabilizing agents. These reducing or denaturing agents can dimerize hα2M in two ways (Figure 7): (i) reducing agents break disulfide bonds between covalently linked monomers, forming dimers through noncovalent forces; (ii) denaturing agents destabilize noncovalent interactions, resulting in disulfide‐linked dimers [56, 80, 82, 83].

Dimers with non‐covalently associated vicinal monomers retain antiproteolytic activity, whereas covalent disulfide‐linked dimers lack this activity [50, 56]. Despite conflicting results for this latter statement [80], it was later demonstrated that the basic unit for antiproteolytic activity is a dimer formed by reducing agents (non‐covalently linked dimer), and that protease binding by disulfide‐linked dimers (e.g., those treated with urea) results from the reassociation of two disulfide‐linked dimers and is not an inherent property of the disulfide‐bound hα2M half‐molecule [54, 56]. This is consistent with the structures presented in Section 3.2 and with the protease capture mechanism described in Section 4.1. These studies of the dimeric form of hα2M provide new insights into the role of this protein in inflammatory processes in vivo and how dynamic hα2M can function.

## 6 Electrophoretic Behavior and Autolysis of the Thioester Bond

In this section, we address the distinctive electrophoretic behavior of hα2M, which is essential for proper interpretation of experimental results involving this molecule, and briefly discuss the diverse abbreviations used for α2M in the literature. Throughout this review, we use the abbreviation α2M to refer to α2‐macroglobulins in general and hα2M to denote the human molecule. The induced conformations are designated as hα2M–MA (methylamine‐induced) and hα2M–protease (protease‐induced). However, the literature employs diverse abbreviations and nomenclatures that may be confusing, particularly for non‐specialist readers. Some of the most common terms used to describe the native form of α2M include native, functional, or active state, as well as *n*α2M. The compacted form of α2M following protease capture or treatment with small amines is commonly referred to as the induced, transformed, activated, or collapsed form. In addition, several acronyms are used in the literature, including *i*α2M, *t*α2M, α2M* (generally referring to primary amine activation, also termed α2M–NH_3_), α2M–MA (methylamine‐induced, the classical amine used in α2M studies), and α2M–protease (protease‐induced). In the latter case, the protease may be abbreviated (e.g., α2M‐T for trypsin or α2M–PPE for porcine pancreatic elastase) [9, 15, 18, 81, 84, 85, 86]. These terms may refer to either human α2‐macroglobulin (hα2M) or homologues from other species (e.g., mouse α2M [86]). In some cases, the Greek letter α is replaced by the Latin letter A (A2M) in the literature. Another widely used classification distinguishes the native and induced conformations of hα2M as the “*slow form*” and “*fast form*,” respectively. This nomenclature reflects the increased electrophoretic mobility of the induced form on native gels, resulting from its more compact structure, reduced dimensions, and altered surface charge [3, 11, 50].

**FIGURE 8:**
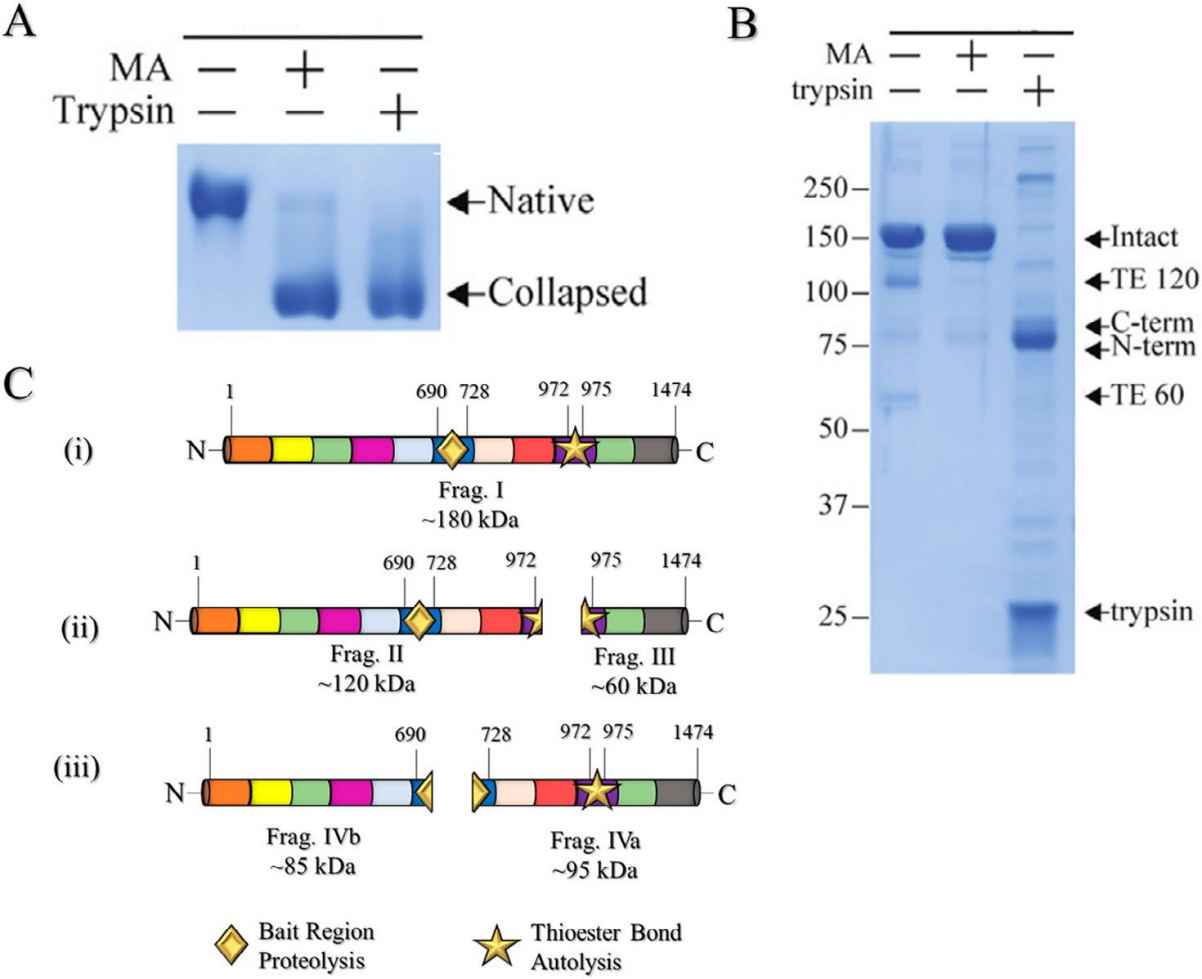
Native PAGE and SDS‐PAGE analysis of the electrophoretic behavior of hα2M and its autolytic fragments. (A) Pore‐limited native PAGE of wild‐type hα2M, treated as indicated with methylamine (MA) and trypsin. Black arrows indicate the native and induced (collapsed) forms. (B) Reducing SDS‐PAGE showing methylamine treatment and proteolysis of hα2M. Black arrows indicate: (i) the autolytic fragments at the thioester (TE) site that appear upon heating under denaturing conditions, labeled TE‐120 and TE‐60, and (ii) the proteolytic fragments resulting from bait region (BR) cleavage, labeled C‐terminal and N‐terminal. (A, B) Reproduced from Harwood et al. [15], with modifications, under CC BY 4.0 license: https://www.jbc.org/article/S0021‐9258(22)00672‐X/fulltext. (C) Schematic representation of hα2M subunits observed in SDS‐PAGE. (i) intact monomer (fragment I, ~180 kDa); (ii) site of thioester autolysis generating heat‐induced fragments (II and III, or TE‐60 and TE‐120, ~60 and ~120 kDa, respectively); and (iii) site of bait region (BR) cleavage separating proteolytic fragments (IVb and IVa, or N‐terminal and C‐terminal, ~85 and ~95 kDa, respectively). The BR site is represented by a gold diamond, and the thioester site is represented by a gold star. Adapted from Barret [3].

To experimentally distinguish between the native and induced conformational states of hα2M, two electrophoretic techniques are particularly useful (i) native polyacrylamide gel electrophoresis (native‐PAGE), performed without reducing agents or heating and typically using a gradient gel (Figure 8A); and (ii) SDS‐polyacrylamide gel electrophoresis (SDS‐PAGE) (Figure 8B), which can be performed with or without disulfide reduction [3, 17, 50]. Native‐PAGE is used to evaluate the two forms of hα2M—native or induced—based on their differences in migration (Figure 8A). However, recent studies report structural changes undetectable by PAGE, suggesting that gel analysis—widely used for decades to assess the native integrity of hα2M—is insufficient for conclusive evaluation. To complement the analysis of the native state, surface hydrophobicity assessment via fluorescence measurements using 4,4′‐Dianilino‐1,1′‐binaphthyl‐5,5′‐disulfonic acid dipotassium salt (bisANS) should be performed [81].

SDS‐PAGE under reducing conditions allows the assessment of the homogeneity of the two different forms. Finally, SDS‐PAGE under non‐reducing conditions enables the evaluation of the integrity of disulfide‐linked dimers [3, 50]. The native form of hα2M from a pure sample displays three bands on an SDS gel after reduction: one at ~180 kDa, corresponding to the intact monomer; one at ~120 kDa; and one at ~60 kDa [17, 87] (Figure 8B, first lane). The latter two fragments result from autolysis of the thioester bond upon heating in the presence of SDS and are referred to by Barrett as Fragments II and III [3]. The abundance of these fragments increases with longer exposure to heat [87]. In addition, it has been reported that the pure native form should not display any components between ~75 and 100 kDa [3, 6, 19, 50, 87]. In a non‐reducing SDS gel, the only difference in the migration pattern of the native form is the appearance of a band at ~360 kDa, corresponding to the disulfide‐linked dimer of hα2M. However, two additional bands at ~120 and ~60 kDa may still be observed, resulting from the autolysis of some monomers [3, 15, 87].

The form induced by methylamine (hα2M–MA), when analyzed by SDS electrophoresis under reducing conditions, displays a single band at ~180 kDa, indicating that it is not susceptible to autolysis of the thioester bond [3]. This is because the bond has already been cleaved, and the reactive carbonyl of Gln^952^ is covalently bound to the amine, preventing fragment separation by heat‐induced autolysis [50] as shown in the second lane of the SDS gel (Figure 8B, second lane). Finally, the protease‐induced form may display multiple bands on SDS electrophoresis under reducing conditions (Figure 8B, third lane).

The initial trap hypothesis suggested that denaturation of the hα2M–protease complex would release the still‐active substrate protease [1, 3]. In some cases, proteases are released in their native state; however, radiolabeling mapping experiments revealed fragments covalently bound to fractions of hα2M monomers and protease subunits. These fragments vary in size depending on the protease type and the number of covalent bonds formed (e.g., one bond per monomer, resulting in high‐molecular‐weight bands > 180 kDa), particularly with large two‐chain proteases such as plasmin, factor Xa, and plasma kallikrein [3, 6]. Several common fragments reported by different authors highlight a main observation in samples complexed with protease analyzed by reducing SDS‐PAGE: the appearance of fragments near ~85 kDa, regardless of the protease class (serine, cysteine, aspartic, or metalloprotease) [20, 87, 88]. These fragments are similar in size (~85 and ~95 kDa) and correspond to the N‐terminal and C‐terminal regions of the hα2M monomer, respectively, cleaved near the central portion of the BRD. The fragments were designated IVa and IVb by Barrett (Figure 8B, third lane), and their detection under denaturing and reducing conditions indicates that proteolytic cleavage occurred in the BR [3, 19, 20].

The fragments mentioned above are best understood in the schematic representation (Figure 8C):

- Fragment I represents the 180 kDa monomer.
- Fragments II and III correspond to autolysis products resulting from thioester bond cleavage, with approximate molecular masses of ~120 and ~60 kDa, respectively—although their apparent sizes may vary depending on gel percentage and molecular weight marker used (e.g., Sigma SRP6314).
- Finally, fragments IVa and IVb (~95 and ~85 kDa, respectively) are generated by reaction with the substrate protease—depending on gel percentage, they may appear as a single band near ~85 kDa [15, 20, 87, 88].

## 7 Thioester Bond Reformation and Covalent Complexes of Non‐Proteolytic Ligands

Having outlined the structural features of hα2M, its protease capture mechanism, and the key concepts required to interpret experimental studies, we now turn to a distinctive mechanism that emerges from these structural and mechanistic principles, involving the covalent incorporation of non‐proteolytic ligands into the internal thioester bond in vitro. Although there is currently no evidence that this process occurs in vivo for tetrameric hα2M, a mechanistically similar process may occur in monomeric α2M homologues, in which the thioester bond is more exposed (e.g., during the conversion of C3 to C3b, followed by the covalent attachment of C3b to diverse biological surfaces [89]). In this broader context of ligand interactions, the most frequently reported in vivo interactions involve the binding of hα2M to various cytokines and growth factors [4, 7, 8, 18]. Numerous studies have investigated these interactions, and although some ligands interact with hα2M in its native form, most cytokines and growth factors preferentially bind to activated hα2M, thereby preventing proteolysis or modulating their activities [8, 10, 25]. These interactions can be covalent or non‐covalent, occur at different sites on the hα2M molecule, and may also be reversible [4, 8].

Beyond these interactions, some studies have examined the incorporation of non‐proteolytic ligands into hα2M in vitro. Grøn and Pizzo [59] described a protease‐independent mechanism that enables the incorporation of ligands ranging from small peptides (as short as 9 amino acids) to proteins of up to 120 kDa [60, 90]. In addition to this pathway, non‐proteolytic ligands can also be co‐incorporated during protease attack, through covalent binding to one of the four thioesters cleaved during the protease‐induced conformational change of hα2M, typically yielding 1:1:1 complexes of hα2M, protease, and ligand [84, 91]. This incorporation strategy can be mimicked by nucleophilic activation with primary amines, which offers practical advantages by eliminating the presence of a protease within the internal “prey chamber” of hα2M, thereby permitting the incorporation of a greater number and larger non‐proteolytic ligands. In contrast, protease‐containing complexes are limited by competition for remaining binding sites and by steric constraints imposed by the pre‐incorporated protease, which remains catalytically active and can cleave co‐incorporated proteins, markedly reducing incorporation efficiency [60, 92].

This concept of incorporating binders through chemical activation originated from early studies by Pangburn [92] and Grøn et al. [93], which demonstrated that cleavage of the internal thioester bond can be reversible [93, 94]. Following thioester cleavage and removal of excess ammonia, heating promotes release of the NH_3_ group incorporated into Gln^952^; however, because NH_3_ is a poor leaving group, this process requires elevated temperatures (> 37°C), with larger amine substituents requiring additional energy (e.g., methylamine compared with ammonia) [60]. In the absence of ligands, amine removal allows reformation of the β‐cysteinyl γ‐glutamyl thioester bond between Gln^952^ and Cys^949^, enabling reversion of hα2M monomer to its native conformation, although full reconstitution of all four monomers occurs only in a fraction of molecules [60, 93, 94]. During this process, non‐proteolytic ligands can be incorporated into either Cys^949^ or Gln^952^, depending on ligand properties and reaction conditions [60, 84, 86, 94]. This incorporation is proposed to proceed via a two‐step mechanism, in which heating‐dependent amine release generates a highly reactive, transient “nascent” intermediate (<hα2M>) that is not detectable by electrophoretic methods; subsequent covalent binding of the thioester to the ligand stabilizes the hα2M*–ligand complex and prevents reversion to the native conformation, rendering the process irreversible in the presence of ligands [60, 86, 92, 95]. This mechanism also occurs in interspecies homologues (e.g., mouse α2M) [86].

Several factors influence the incorporation of non‐proteolytic ligands into hα2M via the nucleophilic substitution mechanism, such as pH, temperature, ligand concentration, incubation time, and, to a lesser impact, ionic strength [60, 61, 92]. It has been reported that, using this mechanism, 5%–15% of antigens could be incorporated into hα2M–NH_3_, enhancing their delivery to the immune system [90]. This results in increased antibody production and cytotoxic T lymphocyte responses, with improved MHC‐I and MHC‐II presentation to T cells, significantly elevating antibody titers compared to other adjuvants [92, 95].

## 8 Purification and Storage of Human α2‐Macroglobulin

Human blood is a complex mixture of biomolecules, including a wide variety of proteins with distinct biological functions, which makes the purification of specific proteins at high purity levels particularly challenging [3, 17, 96]. The earliest reports of hα2M separation emerged from Cohn's ethanol fractionation procedure for human plasma [2, 5, 96], as previously mentioned. Since then, several methods for purifying hα2M have been explored. Here, we aim to highlight key considerations for obtaining hα2M in its native form while providing an overview of the purification strategies employed. Human macroglobulin is present in both serum and plasma; however, due to its strong propensity for protease trapping, the use of serum is not recommended. Proteases involved in coagulation typically activate hα2M, resulting in its induced form in this fluid [3, 17]. Furthermore, it is essential to use freshly collected blood or plasma [3, 17].

Recently, a discussion has arisen regarding the use of non‐frozen fresh plasma (NF) versus frozen fresh plasma (FP) [85, 97, 98]. Nevertheless, frozen fresh plasma has been employed for decades by various research groups, and it has been demonstrated that the macromolecular environment of plasma—rich in diverse molecules—exerts a cryoprotective effect that preserves the native state of proteins during freezing [3, 17, 85]. hα2M purified from both NF and FP has been shown to exhibit comparable properties and structure, maintaining its biological activity [85]. This facilitates the purification process, which would otherwise be more challenging if only fresh plasma were used. Nevertheless, some precautions must be taken when using frozen plasma, such as thawing at 37°C in a water bath. Thawing at room temperature or at 4°C can result in protein precipitation and activation of the intrinsic coagulation pathway. In addition, freeze–thaw cycles should be avoided [3, 17]. Importantly, hα2M isolated from frozen plasma displays no significant differences in biochemical properties or functional activity, regardless of whether the plasma was stored frozen for a few months or for up to 3 years; however, data beyond this storage period are not available [85].

In the initial stages of hα2M purification, protease inhibitors are commonly added [11, 17, 19, 85], and procedures are typically carried out rapidly at 4°C to prevent activation of plasma proteases and coagulation factors [3, 17, 19]. Additional precautions include using plastic or silicone‐coated glassware, as untreated glass surfaces can enhance protease activity via Hageman factor–dependent pathways [19]. Many protocols start with a precipitation step to remove a large fraction of contaminants, most commonly using polyethylene glycol (PEG) or ammonium sulfate ((NH_4_)_2_SO_4_) [2, 3, 17, 53, 85, 96, 99]. Although ammonium sulfate precipitation is a classical method, it has largely been abandoned because amines can alter the native conformation of hα2M [3, 96]; some studies still employ it with careful pH control [96]. Alternative strategies using only PEG precipitation combined with ethacridine monolactate monohydrate (Rivanol) have also been reported, though its use in plasma has been banned in some countries [96, 100].

Subsequent purification steps often involve combinations of chromatographic techniques, including gel filtration, immunoadsorbent chromatography, and Cibacron Blue sepharose chromatography, with variable yields [3, 17, 19, 96, 99]. Many approaches required plasma from donors with haptoglobin type 1–1, as separation from polymeric haptoglobin was challenging [3, 96]. This limitation was largely overcome with metal affinity chromatography introduced in the late 1970s, particularly using zinc‐loaded columns, which exploit the ability of hα2M to bind zinc [26], eliminating the need for selected plasma sources and improving contaminant removal [17, 99]. Despite these advances, large‐scale methods achieving high yield, low cost, and preservation of native hα2M structure remain limited [96]. Current methods generally involve four main steps: (i) PEG precipitation, (ii) immobilized metal affinity chromatography (IMAC) on zinc, (iii) ion exchange chromatography (IEC), and (iv) gel filtration [11, 14, 15, 51, 53, 85].

To conclude this section, it is important to emphasize the storage conditions of purified hα2M, as the molecule readily loses its native conformation during prolonged storage if not handled appropriately, leading also to the formation of high‐molecular‐weight aggregates [3, 17, 81, 85]. The protein should be stored in small aliquots to avoid repeated freeze–thaw cycles [3, 17], using buffers at near‐physiological pH [3, 17], without the addition of sodium azide [81], and containing a cryoprotectant, even during rapid freezing at temperatures below −20°C [81]. Sucrose is more effective than glycine as a cryoprotectant in both freezing protocols and lyophilization, a process that alters hα2M structure by disrupting hydrophilic interactions [17, 81]. Additionally, protease inhibition assays can quantify the antiproteolytic activity of purified hα2M, thereby estimating the fraction of native species [3, 17, 51]. Proper storage is therefore essential to preserve the native conformation and biological activity of hα2M, which is highly sensitive to freezing and lyophilization [81, 85]. Such preservation is crucial for ensuring the validity of subsequent functional studies of this protein.

## 9 Future Perspectives and Limitations

In recent decades, advances in biotechnology, protein engineering, and recombinant expression systems have enabled the production of recombinant hα2M [51, 52], providing a viable alternative to fresh blood or plasma, which are often prioritized for other essential applications, such as the manufacture of human serum albumin, immunoglobulins, and coagulation factors [96]. Importantly, engineered hα2M variants can enable selective capture of specific proteases or ligands and support the design of tailored delivery platforms, expanding the therapeutic and investigative potential of hα2M‐based systems [51, 53].

In this context, synthetic biology approaches have demonstrated the feasibility of neutralizing or delivering defined targets through cells expressing LRP1 or CS‐GRP78 (e.g., neutralization of TNF‐α using recombinant RBD units in osteoarthritis and myocardial infarction models) [37]. Notably, CS‐GRP78 has been identified as a potential target in high‐risk cancers [67, 69, 72], further supporting exploration of its interaction with hα2M. In parallel, the chaperone activity of hα2M, mainly characterized in vitro [27, 73, 74], may have relevant physiological roles, including antigen presentation during infection, transport of circulating molecules or drugs, or prevention of protein aggregation [27, 28, 101, 102]. In line with these observations, initial studies suggest a potential anticancer effect of hα2M in melanoma, where metalloproteinases drive tumor progression [103]. In this broader context, translational studies further support the therapeutic relevance of the purified hα2M, as recombinant hα2M or platelet‐poor plasma (PPP)–hα2M‐rich preparations have demonstrated potential therapeutic effects in cell‐based and preclinical in vivo models, as well as in patients with cartilage pathology, musculoskeletal pathology, and inflammatory arthritis [33, 34, 36, 37]. Consistently, PPP–hα2M‐rich formulations have been tested in patients for neuropathic pain and degenerative disc disease (DDD) fibronectin–aggrecan complex (FAC)–positive, with favorable outcomes reported [32, 35, 104, 105].

Nevertheless, large‐scale clinical trials are still required; moreover, PPP formulations contain multiple bioactive factors (e.g., PDGF‐BB, TGF‐β1, VEGF, and bFGF), which prevents the attribution of the observed therapeutic effects solely to hα2M [105]. Moreover, some in vivo functions of hα2M remain poorly understood, partly due to the rapid clearance of its induced forms (e.g., the functional implications of cytokine and growth factor interactions and the associated downstream hα2M–ligand–cell response pathways) [8]. Although hα2M knockout models have been available since the late 1990s, they have been used in only a few studies of disorders, such as acute pancreatitis [18, 106]. Finally, as discussed in Section 8, commercially available lyophilized hα2M may be unsuitable for certain applications, particularly structural studies, and cost‐effective, high‐yield purification strategies remain lacking [81, 96].

## 10 Conclusion

In this review, we summarize key structural, mechanistic, and functional aspects of hα2M, a broad‐spectrum protease inhibitor extensively studied across multiple research fields. Recent cryo‐EM studies have provided important insights into the dynamics of protease capture and conformational transitions, indicating that activation occurs as a continuous process [10, 11, 12]. The plug‐in‐channel hypothesis, which may explain the initiation of monomer conformational change, is also discussed [13, 15, 53]. Amine‐induced hα2M retains partial antiproteolytic activity in vitro [48, 49, 50], and the noncovalent dimer emerges as the minimal functional unit for protease capture, an aspect often overlooked. We further highlight characteristic electrophoretic profiles of native, induced, autolyzed, and protease‐cleaved forms of hα2M, which are critical for correct experimental interpretation [3, 50, 87], as well as current challenges in analyzing native hα2M beyond conventional native‐PAGE [81]. We address non‐proteolytic ligand incorporation, thioester bond reformation, and plasma storage conditions relevant to hα2M preservation [81, 85], and discuss current research limitations and future directions in the field.

Although an extensive body of literature on hα2M has accumulated over the past five decades, a comprehensive coverage of all aspects was beyond the scope of this review. Readers are therefore encouraged to consult the cited primary literature and other detailed reviews [3, 4, 7, 8, 10, 11, 12, 13, 18, 19, 50]. As an evolutionarily conserved protein [4, 71], hα2M plays a critical role in immune responses and is present in diverse biological systems. Studying this molecule may reveal previously unrecognized aspects of innate immunity and facilitate the development of new therapies, advance understanding of infectious processes, contribute to cancer research, biomarker discovery for metabolic disorders, and other areas highly relevant to human health.

## Author Contributions

All authors contributed equally to this work.

## Funding

This work was supported by Funda ção de Amparo à Pesquisa do Estado de São Paulo (FAPESP) 2022/02456‐0, Coordena ção de Aperfei çoamento de Pessoal de Nível Superior (CAPES) 001, Conselho Nacional de Desenvolvimento Científico e Tecnológico (CNPq) 423532/2018‐9.

## Conflicts of Interest

The authors declare no conflicts of interest.

## Data Availability

Data sharing not applicable to this article as no datasets were generated or analyzed during the current study.
